# The Stealthy Superbug: the Role of Asymptomatic Enteric Carriage in Maintaining a Long-Term Hospital Outbreak of ST228 Methicillin-Resistant *Staphylococcus aureus*

**DOI:** 10.1128/mBio.02039-15

**Published:** 2016-01-19

**Authors:** Laurence Senn, Olivier Clerc, Giorgio Zanetti, Patrick Basset, Guy Prod’hom, Nicola C. Gordon, Anna E. Sheppard, Derrick W. Crook, Richard James, Harry A. Thorpe, Edward J. Feil, Dominique S. Blanc

**Affiliations:** a Hospital Preventive Medicine Service, University Hospital of Lausanne, Lausanne, Switzerland; b Institute of Microbiology, University Hospital of Lausanne, Lausanne, Switzerland; c NIHR Oxford Biomedical Research, John Radcliffe Hospital, Oxford, United Kingdom; d Department of Physics and Centre for Networks and Collective Behaviour, University of Bath, Bath, United Kingdom; e Department of Biology and Biochemistry, University of Bath, Bath, United Kingdom

## Abstract

Whole-genome sequencing (WGS) of 228 isolates was used to elucidate the origin and dynamics of a long-term outbreak of methicillin-resistant *Staphylococcus aureus* (MRSA) sequence type 228 (ST228) SCC*mec* I that involved 1,600 patients in a tertiary care hospital between 2008 and 2012. Combining of the sequence data with detailed metadata on patient admission and movement confirmed that the outbreak was due to the transmission of a single clonal variant of ST228, rather than repeated introductions of this clone into the hospital. We note that this clone is significantly more frequently recovered from groin and rectal swabs than other clones (*P* < 0.0001) and is also significantly more transmissible between roommates (*P* < 0.01). Unrecognized MRSA carriers, together with movements of patients within the hospital, also seem to have played a major role. These atypical colonization and transmission dynamics can help explain how the outbreak was maintained over the long term. This “stealthy” asymptomatic colonization of the gut, combined with heightened transmissibility (potentially reflecting a role for environmental reservoirs), means the dynamics of this outbreak share some properties with enteric pathogens such as vancomycin-resistant enterococci or *Clostridium difficile*.

## IMPORTANCE

Using whole-genome sequencing, we showed that a large and prolonged outbreak of methicillin-resistant *Staphylococcus aureus* was due to the clonal spread of a specific strain with genetic elements adapted to the hospital environment. Unrecognized MRSA carriers, the movement of patients within the hospital, and the low detection with clinical specimens were also factors that played a role in this occurrence. The atypical colonization of the gut means the dynamics of this outbreak may share some properties with enteric pathogens.

## INTRODUCTION

Hospital-associated methicillin-resistant *Staphylococcus aureus* (HA-MRSA) remains a key public health challenge. In addition to prevention policies, effective programs of infection control rely on early detection and effective containment. However, hospital outbreaks of HA-MRSA remain commonplace, and new outbreak clones emerge regularly (1–3). The underlying risk factors are both extrinsic (i.e., relating to infection control procedures, opportunities for transmission, and the hospital environment) and intrinsic (i.e., relating to the properties of the bacteria). In terms of extrinsic factors, many aspects of the hospital setting can impact transmission, including compliance with infection control measures, patients’ comorbidities, bed occupancy, and the number of nurses per patient (4, 5). As for intrinsic factors, strains have been identified by a heightened ability to be transmitted on national (6) and/or international (7) scales, although the genetic basis of this trait is poorly understood (8, 9).

Classical molecular typing such as pulsed-field gel electrophoresis or targeted sequence-based methods (e.g., *spa* typing, double locus sequence typing [DLST]) have been used for the last three decades to investigate outbreaks and are described as “highly discriminatory.” However, isolates from a predominant clone obtained in different countries could often not be differentiated; thus, it was not possible to infer whether outbreaks arose through multiple introductions of the predominant clone or from a single point source. Whole-genome sequencing (WGS) promises to make a significant contribution to infection control through unambiguously defining outbreak isolates within a single hospital and even potentially providing the means to reconstruct transmission pathways (10–14). However, current studies have focused on short-term outbreaks persisting over the course of several weeks or months. In contrast, our tertiary care hospital experienced a 4-year outbreak between 2008 and 2012 that affected over 1,600 patients and was due to the HA-MRSA sequence type 228 (ST228) SCC*mec* I clone (9). This clone is common in central Europe, is also called the southern German or Italian clone, and has been present in our hospital since the year 2000 (15). Classical epidemiological typing was not sufficiently powerful to determine if this outbreak was due to the dissemination of a single strain or if it resulted from multiple introductions. Another objective was to reconstruct the transmission pathways within the hospital in order to help optimize future infection control strategies. In the present study, we characterize the course of this outbreak by combining whole-genome sequence data with detailed epidemiological and clinical data. Our analyses point to a role of intrinsic properties of the clone, specifically a propensity for asymptomatic carriage and heightened transmission, in maintaining the outbreak.

## RESULTS

### Description of the outbreak.

**FIG 1:**
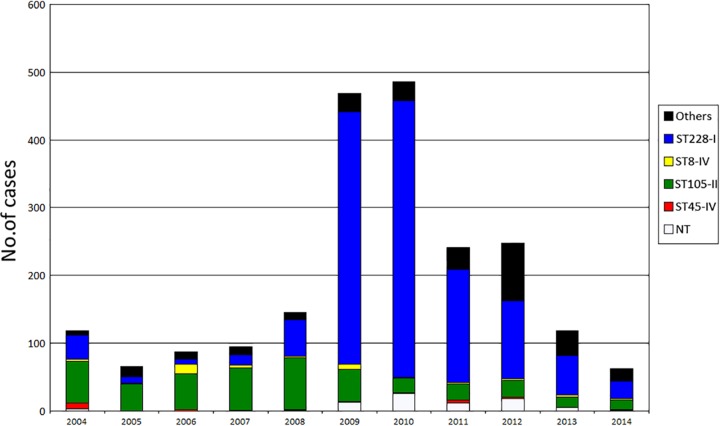
Annual incidence of MRSA cases in the University Hospital of Lausanne according to sequence type (ST) during the period 2004 to 2014. NT, not typed.

Before the outbreak (2005 to 2007), the mean incidence of newly identified MRSA patients per 1,000 admissions was 8.1. Half of them were identified more than 48 h after admission, among whom 63% harbored the predominant clone ST105-SCC*mec* II. Since September 2008, an increasing number of new cases was observed, most belonging to ST228-I. The highest incidence was reached in April 2010, and the incidence then decreased (Fig. 1). The annual number of cases per 1,000 admissions increased to 26.6 in 2010, among which 73% were identified ≥48 h after admission (hospital onset). In parallel, from 2007 to 2010, the proportion of MRSA strains among clinical *S. aureus* isolates increased from 12% to 23%, and the annual number of MRSA bacteremia increased from 20 to 46 episodes. A low-level resistance (LLR) to mupirocin (mean MIC, 25 mg/liter) was identified as a distinguishing phenotype of this epidemic strain. This resistance was rarely found in other clones and thus provided a useful marker to monitor the outbreak.

### Reinforcement of infection control measures.

This outbreak led to reinforcement of infection control measures, including recurrent information and training and observations of health care workers (HCWs). Procedures for decontamination of the patient’s environment were reinforced, and additional staff was dedicated to this task. A PCR-based rapid screening test for MRSA was introduced in June 2009, allowing reduction in mean turnaround time to 6.75 h from 57 h for negative cultures and 85 h for positive culture (data not shown). This rapid test was used for screening roommates and all patients admitted to or leaving the intensive care units (ICUs). When 2 or more new cases were observed in the same unit over the course of a single week, weekly screenings by culture of all patients were performed until 2 weeks passed without any new cases. In addition, all patients leaving such a unit were screened by PCR. Patients in units hosting 2 or more MRSA carriers were screened on a weekly basis. Since the introduction of this measure (October 2010), a marked decrease in new cases has been observed (Fig. 1).

### Screening of HCWs.

In order to identify any putative super spreaders among HCWs, intensive screenings were performed during the first part of the outbreak (September 2008 to January 2009). A total of 511 HCWs were screened, among whom 17 (3.3%) carried MRSA. Only one harbored an isolate belonging to the ST228 clone that expressed LLR to mupirocin. This person was found positive in January 2009, followed one decolonization procedure, and was considered decolonized after three consecutive negative screening cultures at intervals of several weeks.

### Transmissibility.

From January to May 2009, a systematic review of roommate screenings following the detection of new MRSA patients showed a significantly higher proportion of positive roommates with the ST228 clone (24/216 [11.1%]) than was the case with the formerly predominant ST105-II clone (8/116 [6.9%]; *P* = 0.002).

### Sites of colonization.

From January to May 2009, results of screening sites of 184 new carriers were reviewed (114 with ST228, 70 with other clones). Groin samples were significantly more often positive (80.7%) in patients harboring the ST228 clone than in patients with other clones (50%; *P* < 0.0001). This led us to investigate colonization of the gut by MRSA. Between October 2009 and February 2010, a rectal swab was performed in 201 new MRSA carriers with their approval. Among ST228 carriers, 70% (113/162) had positive rectal swabs, compared to 49% (19/39) for carriers of the other clones (*P* = 0.01).

### Whole-genome sequencing.

Two hundred twenty-eight of the 237 isolates selected for WGS analysis were successfully sequenced. Mobile genetic elements (MGEs) were removed from the alignment, to leave only the core genome (*n =* 2,348 genes). A total of 1,565 single nucleotide polymorphism (SNP) positions were recorded among all ST228 isolates from this study. Among these, 1,250 were in genes (316 synonymous, 892 nonsynonymous, and 42 nonsense). The SNPs were evenly distributed throughout the genes, with the exception of five genes showing a significantly higher number of positions (see Text S1 in the supplemental material). Among these were two genes (*fnbB* and *clfB*) coding for surface proteins involved in pathogenicity and virulence.

### Investigation of the beginning of the outbreak.

**FIG 2:**
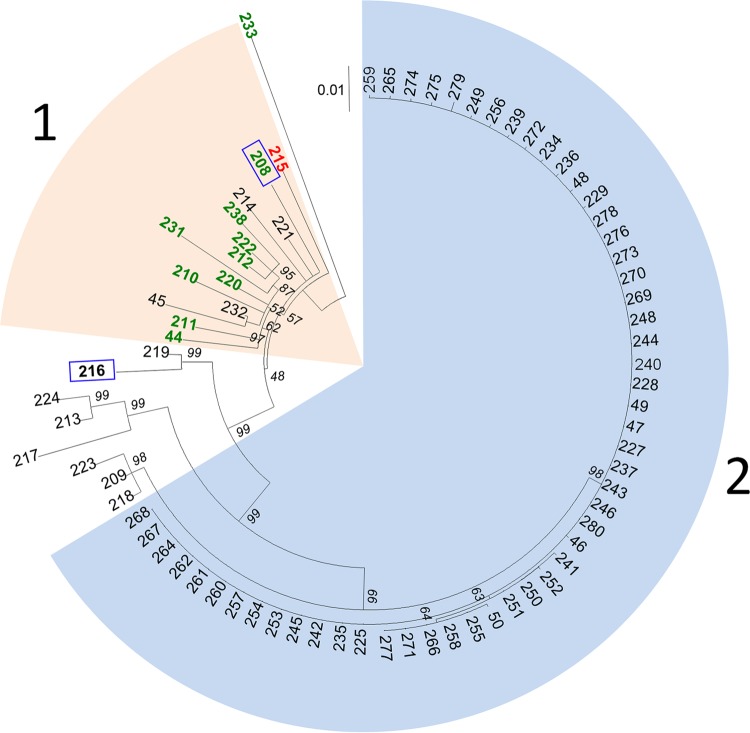
Phylogenetic evidence of a clonal spread of ST228 isolates in 2008 in the University Hospital of Lausanne. Shown is a maximum likelihood tree based on SNP variable sites of ST228 isolates (1 per patient), annotated with the isolate number. The scale bar represents the number of substitutions per SNP site. Most isolates in cluster 1 were collected before the outbreak or were from readmitted patients previously known to be MRSA carriers. Isolates within cluster 2 showed very low diversity (0 to 3 SNPs) suggesting direct transmission. Isolates susceptible to mupirocin are in green, those with low-level resistance to mupirocin (base mutation G→T in the *ileS* gene) are in black, and those with high-level resistance (presence of the *mupA* gene) are in red. Isolates showing the absence of the *qac* gene are surrounded by a blue square. Bootstrap values are shown in italic below each branch.

In 2008, 80 patients were colonized or infected with ST228. Isolates were available for 76 of them and were analyzed by WGS. The maximum likelihood tree of these isolates reconstructed from the core SNPs discriminated two major clusters (Fig. 2). Most isolates in cluster 1 were collected before the outbreak or were from readmitted patients previously known to be MRSA carriers. The diversity within this cluster was relatively high (pairwise SNP differences: mean, 35; range, 11 to 56), with no evidence of direct transmission between patients from whom these isolates were recovered. Conversely, isolates within cluster 2 showed very low diversity, with a maximum of 3 SNP differences. Between these 2 clusters, intermediate isolates could be observed. One outlier (no. 233) (Fig. 2) was isolated from a patient transferred from a hospital in Macedonia, a country where ST228 is known to be prevalent (15).

WGS data confirmed the outbreak started with the index case on 3 September, as no patient was found to harbor an isolate from cluster 2 before this date. The outbreak started in the septic surgery ward. After the first patient was identified, 7 additional patients were found positive in this ward. These 8 patients had been hospitalized for 14 to 45 days prior to MRSA identification and were therefore unrecognized carriers with many opportunities for transmission. Epidemiological data did not allow the identification of the source patient.

Given the very low level of diversity within cluster 2 (53 isolates), the WGS data provide powerful evidence as to which isolates were likely to be epidemiologically linked or unlinked to the outbreak. Four isolates did not belong to cluster 2 and were probably epidemiologically unlinked with the outbreak isolates. In contrast, three patients screened on admission, and hence not suspected to be carrying the outbreak variant, harbored isolates that were identical to the index isolate. On further investigation, all three patients had previous hospital stays in units where the epidemic strain was present.

### Network visualization of patients harboring the outbreak clone.

**FIG 3:**
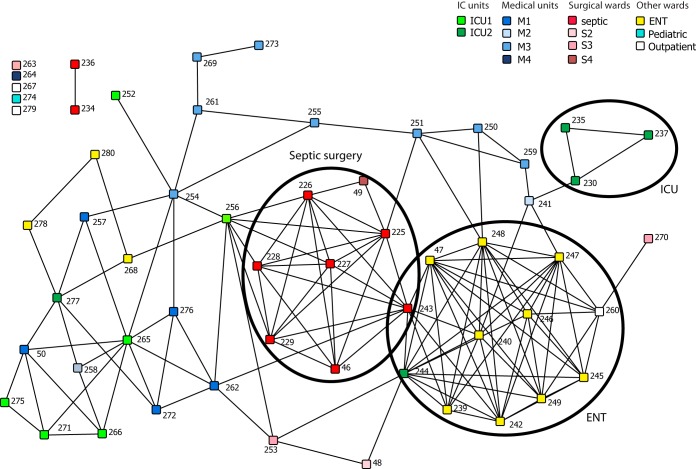
Network visualization of patient movement from September to December 2008. Each node represents a patient, colored by the unit where he or she was hosted when his or her first MRSA was diagnosed. Patients are linked if they spent at least 1 day in the same unit/ward. The layout of the network does not represent geographic location; it is arranged so that well-connected nodes tend to be close to each other. The network clearly shows distinct clusters of well-connected nodes, circled here, corresponding to locations key to this outbreak.

A network analysis based on overlapping periods of hospitalization for each patient harboring an outbreak strain in the early stages of the outbreak (September through December 2008) is presented in Fig. 3. Each square node represents a patient. Pairs of patients are linked if they shared at least 1 day in the same unit/ward. In addition to the 53 patients found to harbor the outbreak strain (i.e., those with isolates in cluster 2), we also included an additional four patients who harbored ST228 isolates for which WGS was not available but were very likely to be part of the outbreak on the basis of DLST data and LLR to mupirocin. This analysis revealed two densely linked groups of patients that correspond to the first (septic surgery [nodes colored red in Fig. 3]) and the third (ear nose and throat [ENT; colored yellow]) units where the outbreak started, whereas the second ward (ICU2 [dark green]) showed only sparse links.

This confirms our hypothesis that the strain had plenty of time to disseminate from the septic surgery and ENT wards. Moreover, the second unit (ICU2) where the outbreak occurred did not seem to have as many links as the other two, indicating that this ICU played a more minor role in the dissemination of the outbreak strain than was considered likely during the course of the outbreak. Notably, for seven patients (12%) there was no clear epidemiological link that could explain acquisition of the outbreak strain based on overlapping stays on a given ward.

### Investigation of the period from 2008 to 2012.

**FIG 4:**
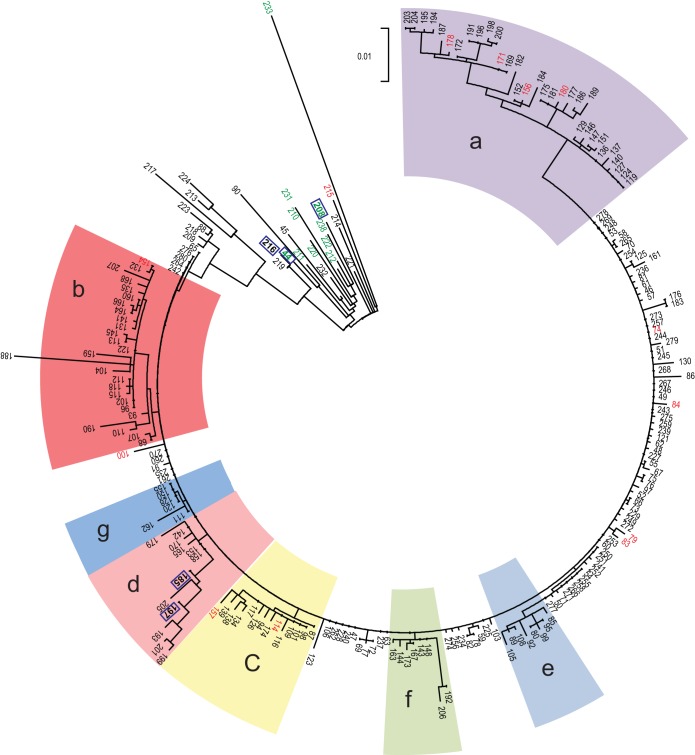
Phylogenetic evidence of the clonal spread of a single variant and its diversification into 7 distinct branches (a to g) over a 52-month period. Shown is a maximum likelihood tree based on SNP variable sites of all ST228 isolates (1 per patient) recovered in 2008 and 1 consecutive patient out of 10 from 2009 to 2012. The scale bar represents the number of substitutions per SNP site. Isolates susceptible to mupirocin are in green, those with low-level resistance to mupirocin (base mutation G→T in the *ileS* gene) are in black, and those with high-level resistance (presence of the *mupA* gene) are in red. Isolates showing the absence of the *qac* gene are surrounded by a blue square.

**FIG 5:**
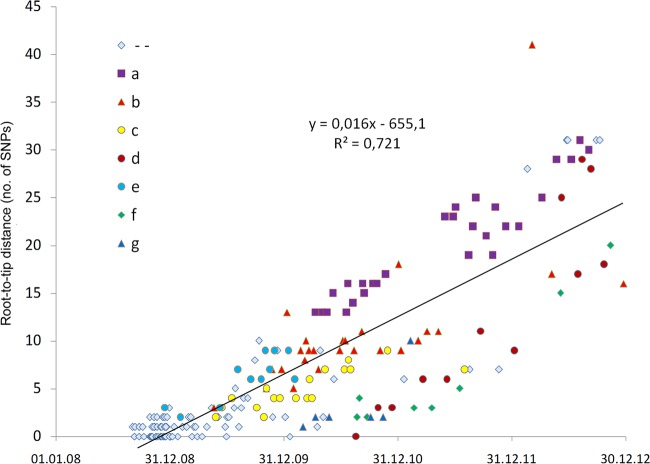
Root-to-tip genetic distances of isolates from the outbreak strain. The total number of SNPs present within each isolate compared to that in the index isolate was computed and plotted against the date on which each isolate was recovered. The color and the shape of the dots are according to the 7 branches observed during the diversification of the strain (Fig. 4).

**FIG 6:**
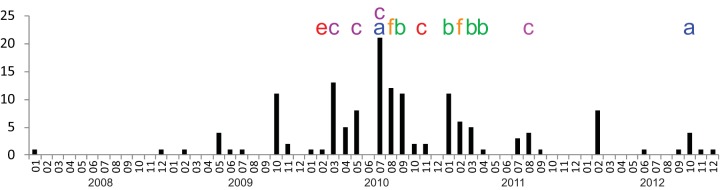
Example of the heterogeneity of isolates from the outbreak strain observed within a single ward. Shown is the incidence of ST228 MRSA patients in the orthopedic ward with indication of patients harboring diversified isolates (a to g) revealed by WGS (Fig. 5).

We then reconstructed a maximum likelihood tree that includes all isolates from 2008 to 2012 (Fig. 4). This analysis confirmed that the whole outbreak was due to the clonal spread of a single variant. During this 52-month period, 7 distinct branches (a to g in Fig. 4 and 5) diversified over time. None of these branches were associated with a specific ward; instead, they were observed in many different wards (see Text S1 in the supplemental material). Figure 6 illustrates the context of the orthopedic ward, where the outbreak lasted several months. During this period, patients with isolates belonging to 5 of these branches were identified, suggesting that at least on five occasions unrecognized MRSA patients were admitted to this ward. WGS data showed that new MRSA patients were not only due to transmissions within the ward but also to admission of unrecognized MRSA patients.

We note that isolate no. 188 is divergent compared to the other isolates of the outbreak and is situated on the end of a long branch (branch b in Fig. 4). While this branch length could be indicative of a hypermutator, we found no mutation in the *mutL* or *mutS* genes involved in hypermutability (10, 16).

### Calculating the rate of diversification of the outbreak clone.

We then examined the rate at which diversification of the ST228 variant accrued over the course of the outbreak. We computed the total number of SNPs present within each cluster 2 isolate compared to the index isolate and plotted this against the date on which each isolate was recovered (Fig. 5). There is a clear positive correlation of genetic divergence with sampling date of isolates, reflecting the accumulation of SNPs over time. The linear regression line (excluding isolate no. 188 [described above]) showed a slope of 0.016 SNP per day (1 SNP every 8.9 weeks). Thus, the finding of two identical isolates from different patients suggests that a transmission event is likely to have occurred at some point within the 9 weeks prior to sampling. Considering the size of the N315 genome (2,814,816 nucleotides [nt]) and the size of subtracted MGE (210,244 nt), a mutation rate of 2.2 × 10^−6^ substitutions per nucleotide site per year was estimated.

### Genetic determinants of resistance of interest.

The WGS data showed that all isolates from cluster 2 showed the G→T mutation in the *ileS* gene conferring LLR to mupirocin (Fig. 2 and 4); thus, this resistance phenotype has been clonally inherited during the outbreak. Fourteen of the 25 (56%) ST228 isolates that did not belong to cluster 2, and hence were epidemiologically unlinked to the outbreak, also exhibited this mutation, although it is rarely observed in other clones. In contrast, the presence of the *mupA* gene, located on a plasmid and responsible for the high-level resistance (HLR), was observed in 14 isolates representing different branches within cluster 2 (Fig. 4). This points to multiple independent acquisitions of the plasmid over the course of the outbreak. All of the outbreak isolates except 5 showed the presence of the *qac* gene by PCR (Fig. 2 and 4).

## DISCUSSION

We combined whole-genome sequencing with detailed metadata to investigate an outbreak of MRSA involving over 1,600 patients in a tertiary care hospital during a 4-year period. To our knowledge, this is the largest-scale outbreak in a single hospital that has yet been characterized using WGS. The WGS data clearly demonstrated that this large outbreak was due to the spread of a single clonal variant of ST228. The genome data are broadly consistent with the epidemiological data in that the earliest outbreak isolate (the index isolate) sequenced was recovered in September 2008, which is when the outbreak began. The data also confirm the septic surgery ward and the ENT ward as the sites where the outbreak originated in the hospital, but network analysis of patient movement suggests that one ICU played a smaller role in propagating the outbreak during the early stages than was assumed at the time.

The identification of an index isolate means that it is possible to calculate the rate of mutation accumulation over the course of the outbreak, simply by plotting the number of SNPs of each outbreak isolate relative to the index isolate against the time of recovery. Our estimate of 2.2 × 10^−6^ SNPs per genome per year is highly consistent with previous estimates (1.3 × 10^−6^ to 3.3 × 10^−6^) (17–20). This equates to an average about 1 SNP every 9 weeks, meaning that the WGS data probably do not have sufficient discrimination to resolve multiple transmission events occurring over shorter time frames than this. The best we can do, therefore, over the short term is to identify groups of patients who cohabited the same ward at overlapping times and harbored identical isolates and infer in such cases that the data are consistent with transmission events having occurred between these patients. It is notable that for 12% of isolates, there is no consistency between patient movement and genome data. The acquisition of the outbreak strain in these cases could therefore have occurred from an HCW (which is not very likely, given that only one HCW was found to be colonized with the isolate) or possibly from an environmental source or unrecognized MRSA patients.

The WGS data for the latter stages of the outbreak confirm the spread of a single clonal variant of ST228. The prolonged outbreak yielded to the diversification of the strain into at least 7 recognizable branches. In most of the wards, isolates from more than one branch were observed. This means that unrecognized MRSA carriers were admitted to these wards and that newly detected MRSA patients were not only due to transmissions within the ward. For instance, in the orthopedic ward, the infection control team did not understand why the spread of the clone could not be controlled. WGS analysis showed that unknown MRSA carriers were probably constantly admitted to this ward (Fig. 6). Movements of such patients must have played a key role in the spread and continuance of this outbreak. This hypothesis is corroborated by the observation of a decline in the outbreak after implementation of a weekly screening of all patients.

Many long-term asymptomatic carriers might have been unrecognized, which would have provided ample opportunities for “stealthy” transmission. Of note, the first eight patients had been hospitalized for 14 to 45 days prior to positive screening. In our setting, the 5-bed rooms were found to be the epicenter of the spread in many wards. Within these rooms, patients shared facilities and had social contacts that could have favored the transmission of the strain. We observed that the infection control strategy was globally insufficient to avoid new transmissions, and several measures were progressively introduced or reinforced. The role of unknown colonized patients is supported by the fact that the fading of the outbreak started after systematic screening was implemented (weekly in units with hospitalized MRSA patients, on ICU admission and discharge, and at discharge in units where the strain was spreading). Indirect transmission via the environment might have also played a role, but we have no data to support this hypothesis. Nevertheless, reinforcement of disinfection of the vicinity of the patients was one of the first control measures that were introduced.

Another characteristic of the strain was its propensity for enteric colonization. To our knowledge, this is the first time such an association has been reported for a specific strain in comparison with others. Among MRSA carriers, the rate of gastrointestinal colonization was reported to vary from 11 to 89% (21–25). Enteric carriage may lead to a greater contamination of the environment, thus favoring transmission (26–28). This feature might have played a role in the spread of our epidemic strain and would explain why the ST228 outbreak clone appeared to be more transmissible between roommates than other hospital-acquired MRSA clones. Moreover, adaptation to enteric carriage would change the dynamics of the outbreak to more closely resemble enteric pathogens such as vancomycin-resistant enterococci or *Clostridium difficile*. Detailed WGS-based studies on *C. difficile* have revealed that only a minority of cases can be explained through direct transmission from other patients, implicating a role for environmental reservoirs.

The low-level resistance (LLR) to mupirocin and the presence of the *qac* gene might also have played a role in the dissemination of the outbreak. LLR is usually the result of base mutations in the native isoleucyl RNA synthetase (*IleS*) gene which belonged to the core genome (29). High-level resistance (HLR) is mostly due to the acquisition of the plasmid-mediated *mupA* gene, which encodes a novel isoleucyl RNA synthetase (30, 31). The widespread use of intranasal mupirocin ointment for the decolonization of MRSA has raised concerns about resistance (32). The association between clinical use of mupirocin and emergence of resistance is not clear. In the present outbreak, next-generation sequencing results showed that the mutation conferring LLR to mupirocin had a clonal spread. In our hospital, this antibiotic was restricted to MRSA decolonization. Thus, the majority of the patients were not exposed before being colonized with MRSA. Conversely, the presence of isolates expressing HLR to mupirocin at the root of cluster 2 and in different branches (Fig. 4) suggests multiple acquisition of the plasmid containing the *mupA* gene during the outbreak.

The *qac* genes not only codes for resistance to quaternary ammonium compounds but also codes for resistance to a broad spectrum of other cationic compounds, such as chlorhexidine, via an efflux-based pump (33). Considering the wide use of chlorhexidine in the hospital, the presence of the *qac* genes in an MRSA clone could contribute to its adaptation in this setting. We previously showed that the *qac* genes in ST228 strains from our hospital were located on a plasmid closely related to the plasmid SAP064A (9), and the present study showed that the majority of ST228 isolates harbored these genes. This suggests that these genes are associated with the ST228 lineage. Together, resistances to mupirocin and chlorhexidine might have played a role in the dissemination of the clone in our hospital.

Several studies reported nosocomial MRSA outbreaks with clear molecular and epidemiological evidence of MRSA transmission from HCWs to patients (34, 35). In our setting, one HCW was found to harbor the ST228 clone. The isolate has not been sequenced, but its LLR to mupirocin (data not shown) is consistent with it being the outbreak variant. Due to confidentiality policy in our hospital, we did not have access to administrative data from the HCW screened for MRSA and could not assess if he/she took care of a limited or a large number of patients. Nevertheless, the fact that decolonization was successful and that the outbreak continued suggest a transient colonization that probably did not play a major role in the dissemination of the epidemic strain.

In conclusion, we showed that this large outbreak was due to the clonal spread of a specific strain with genetic elements adapted to the hospital environment. However, other factors were probably necessary for this occurrence, such as the long period of hospitalization of unrecognized MRSA carriers at the beginning of the outbreak and the low detection with clinical specimens. Under these circumstances, intensifying the screening of hospitalized patients for earlier detection of carriers together with increasing disinfection of the patient environment were the actions that probably had the greatest impact on the outbreak.

## MATERIALS AND METHODS

### Setting.

The University Hospital of Lausanne is a 1,000-bed tertiary care hospital where active screening for high-risk patients was part of the MRSA control strategy. Screening for MRSA was performed by culture, and at least one isolate per patient was genotyped. (For more details, see Text S1 in the supplemental material.)

### Study period.

The outbreak started in September 2008, reached a peak in 2010, and faded in November 2010. The study period was extended from January 2008 to December 2012.

### WGS.

In assembling the strain collection for sequencing, we sampled deeply from the early stages of the outbreak in 2008 and sequenced the first isolate recovered from all patients either infected or shown to be carriers, including new cases and readmitted patients known to carry this clone (*n =* 80). For the period from 2009 to 2012, one isolate from every 10th consecutive patient was selected for WGS analysis, and only new cases were considered (*n =* 157).

Whole-genome sequencing (WGS) was performed as previously described (14) (For more details, see Text S1 in the supplemental material.)

### Detection of resistance genes of interest.

Previous analysis of ST228 isolates with microarrays revealed the presence of *qac* genes (36). These were found to be located on a conjugative plasmid and code for resistance to cationic biocides via a relatively low-specificity efflux pump (33). A PCR assay for this gene was performed on all isolates selected for WGS analysis as previously described (37). The presence of the *mupA* gene, responsible for high-level resistance to mupirocin, was determined by a PCR assay performed as previously described (38). Low-level resistance (LLR) to mupirocin is usually the result of a single-base mutation (G→T) in the native isoleucyl RNA synthetase (IleS) gene. This mutation, situated at position 1173071 in the N315 reference genome, was searched for in all mapped sequences.

### Network analysis of patients’ movements during their hospitalization.

Ward, unit, and room number in and out dates of hospitalization were retrieved from the informatics system of the hospital. Patients were considered to have acquired MRSA after the date of the last negative screening (if applicable), and onward transmission was assumed to be unlikely after the report of the first positive sample as additional contact measures were then taken. These dates were obtained from the laboratory information system. A MATLAB script was written to calculate the number of days each pair of patients spent in the same location having the opportunity to transmit MRSA. The ward was considered the geographical location, except for large wards (medical, surgery, and ICUs), for which the unit was considered. The data matrix was visualized using the software NetDraw version 2.139 (39).

## SUPPLEMENTAL MATERIAL

Text S1  Supplemental methods (infection control policy for MRSA, microbiology and molecular typing, whole genome sequencing) and supplemental results (SNPs distribution of SNPs across genes, figure of the incidence of ST228 MRSA patients in different wards). Download Text S1, PDF file, 0.1 MB
