# Supplementary material for: The Stealthy Superbug: the Role of Asymptomatic Enteric Carriage in Maintaining a Long-Term Hospital Outbreak of ST228 Methicillin-Resistant Staphylococcus aureus
Source: mBio. 2016 Jan 19;7(1):e02039-15. doi: 10.1128/mBio.02039-15 (PMC4725017; doi:10.1128/mBio.02039-15)
Supplement: Text S1 — Supplemental methods (infection control policy for MRSA, microbiology and molecular typing, whole genome sequencing) and supplemental results (SNPs distribution of SNPs across genes, figure of the incidence of ST228 MRSA patients in different wards). Download [file mbo006152606s1.pdf]

The stealthy superbug: The role of asymptomatic enteric carriage in maintaining a long-term hospital outbreak of ST228 methicillin resistant *Staphylococcus aureus*

Laurence Senn, Olivier Clerc, Giorgio Zanetti, Patrick Basset, Guy Prod'hom, Nicola C. Gordon, Anna E. Sheppard, Derrick W. Crook, Richard James, Harry A. Thorp[5], Edward J. Feil, Dominique S. Blanc

Supplementary material

Infection control policy for MRSA

This included admission screening of patients transferred from a foreign hospital or a nursing home, roommates of newly identified MRSA infected/colonized patients and former carriers at readmission. Routine screening included nose, throat and groin swabs. Wounds, urine (if bladder catheter in place) and infected sites were added if applicable. When two or more roommates were positive, all patients from the unit were screened. Dependent on the results, patients from the whole ward and possibly health-care workers (HCWs) (nasal and throat swabs) were subsequently also screened.

For all patients, standard precautions were applied, which included alcohol-based hand-rub hand hygiene policy based on World Health Organization guidelines. Routine evaluation of compliance with hand hygiene policy was performed as already described (1) and showed a constant progression over the previous years. Contact precautions were added for known MRSA carriers (which include single room or cohorting MRSA patients in the same room), and decolonization was attempted with a 5-day long combination of nasal mupirocin, mouth and skin application of chlorhexidine. A systemic treatment, usually with rifampin and co-trimoxazol, was added in case of urinary colonization.

Microbiology and molecular typing

Screening of MRSA was performed using enrichment broth and chromogenic medium as previously described (2). Susceptibility patterns to antibiotics were used as first line typing results as previously described (3). Susceptibility to mupirocin was determined using Etest (bioMérieux, France); a low level of resistance (LLR) was defined when the minimum inhibitory concentrations (MICs) was between 8 and 64 μg/mL, and high-level resistance with a MICs >512 μg/mL (4).

Genotyping of all patient isolates was carried out using double-locus sequence typing (DLST), the detection of the Panton-Valentine leukocidin (*pvl*) toxin genes and determination of the SCC*mec* type (5). Isolates showing allele 4 for *clfB* or *spa* (DLST4-4, x-4 and 4-x), with a SCC*mec* type I, and the absence of PVL were considered as belonging to the ST228 clone (6) and selected for WGS.

Whole genome sequencing (WGS)

DNA was extracted and sequenced using the Illumina HiSeq 2000 platform (San Diego, CA, USA) as previously described (7). Reads were mapped to reference genome N315, which is the closest sequenced genome to ST228 (both belong to CC5), using Stampy v1.0 (8). Single nucleotide polymorphisms (SNPs) were called across non-repetitive sites using SAMtools mpileup, excluding mobile genetic elements (MGEs) (9). From these SNPs, pair-wise distance matrix and Maximum-Likelihood trees were constructed using MEGA6 (10), with 1000 bootstrap replications.

SNPs distribution of SNPs across genes

We carried out a monte-carlo simulation to statistically evaluate the distribution of SNPs across genes. We concatenated the core genes together, and randomly distributed 1250 SNPs across the concatenated sequence. We then counted the number of SNPs in each gene, and repeated this process 100,000 times. We derived a one-tailed p-value for each gene by dividing the number of times the simulated number of SNPs was greater than or equal to the observed number (e.g. if this criteria was met in 1 simulation, then p = 1/100,000). The p-values were ranked, and a Bonferroni-Holm correction was used to correct for multiple testing.

Table S1. Genes with a significant higher number of SNps (*P* value <0.05)

Gene	Product	Position on N315	Observed	SNPs positions on N315	Simulation average	Simulated_ higher*	*P* value
SA1839	hypothetical protein, similar to SdrH	2075260 - 2076507	11	2076004, 2076016, 2076018, 2076022, 2076028, 2076031, 2076034, 2076043, 2076058, 2076096, 2076098	0.71708	0	0
murZ	UDP-N-acetylglucosamine 1-carboxylvinyl transferase 2	2174362 - 2175621	7	2174720, 2174757, 2174904, 2175130, 2175339, 2175482, 2175575	0.72374	0	0
SA2311	hypothetical protein, similar to NAD(P)H-flavin oxidoreductase	2596052 - 2596723	7	2596130, 2596233, 2596236, 2596338, 2596440, 2596473, 2596491	0.3864	0	0
fnbB	fibronectin-binding protein homolog	2568323 - 2571208	11	2569568, 2569569, 2569578, 2569591, 2569659, 2569682, 2569688, 2569703, 2569721, 2569762, 2569766,	1.65259	0.00001	0.023
clfB	Clumping factor B	2718295 - 2720928	9	2718994, 2719000, 2719018, 2719024, 2719030, 2719042, 2719120, 2719460, 2719817	1.40491	0.00001	0.023

*, equal to or greater than observed.

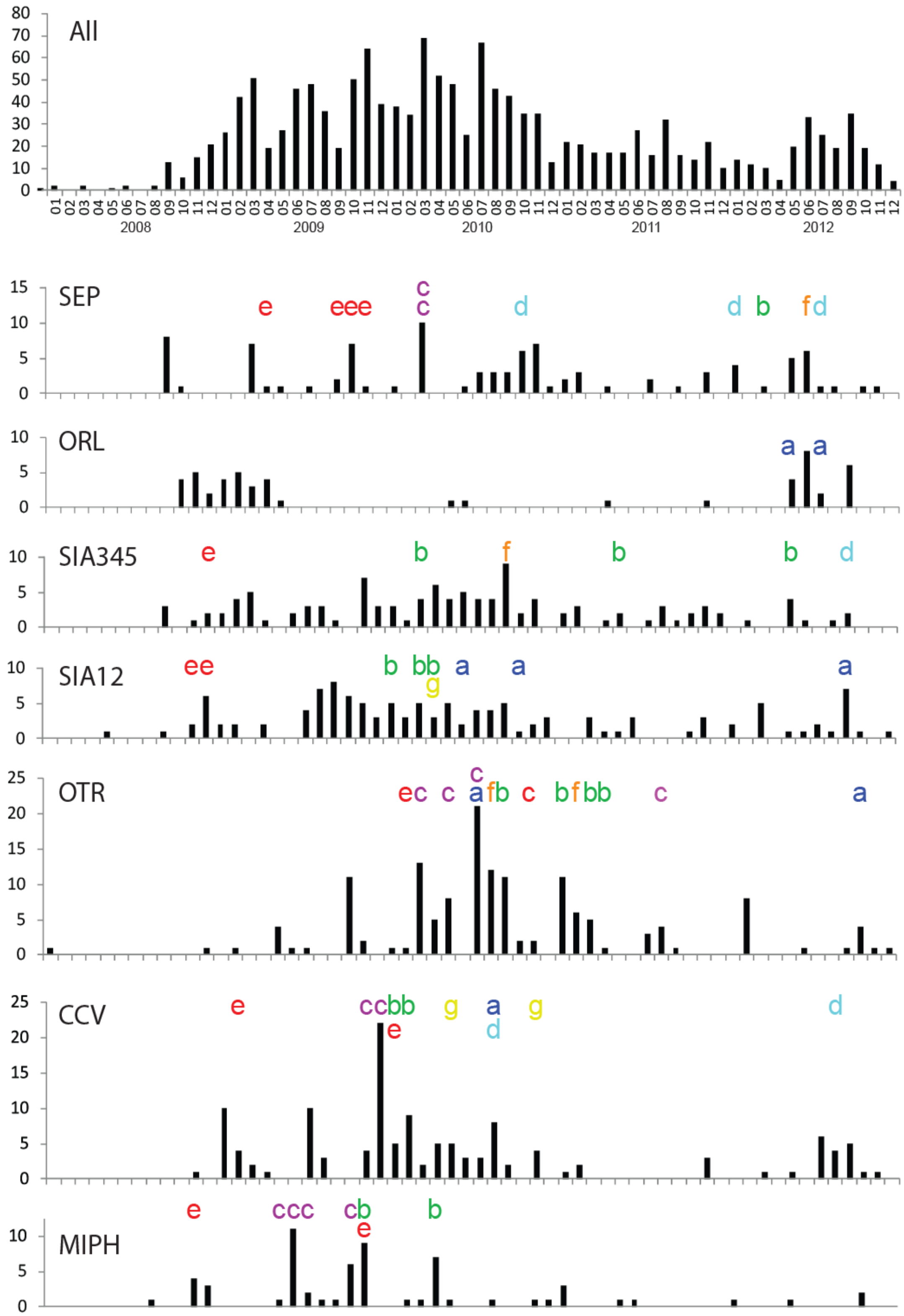

Figure S1. Incidence of ST228 MRSA patients in the different wards with indication of patients harbouring divergent branches (a to g) revealed by WGS (see Figure 5).

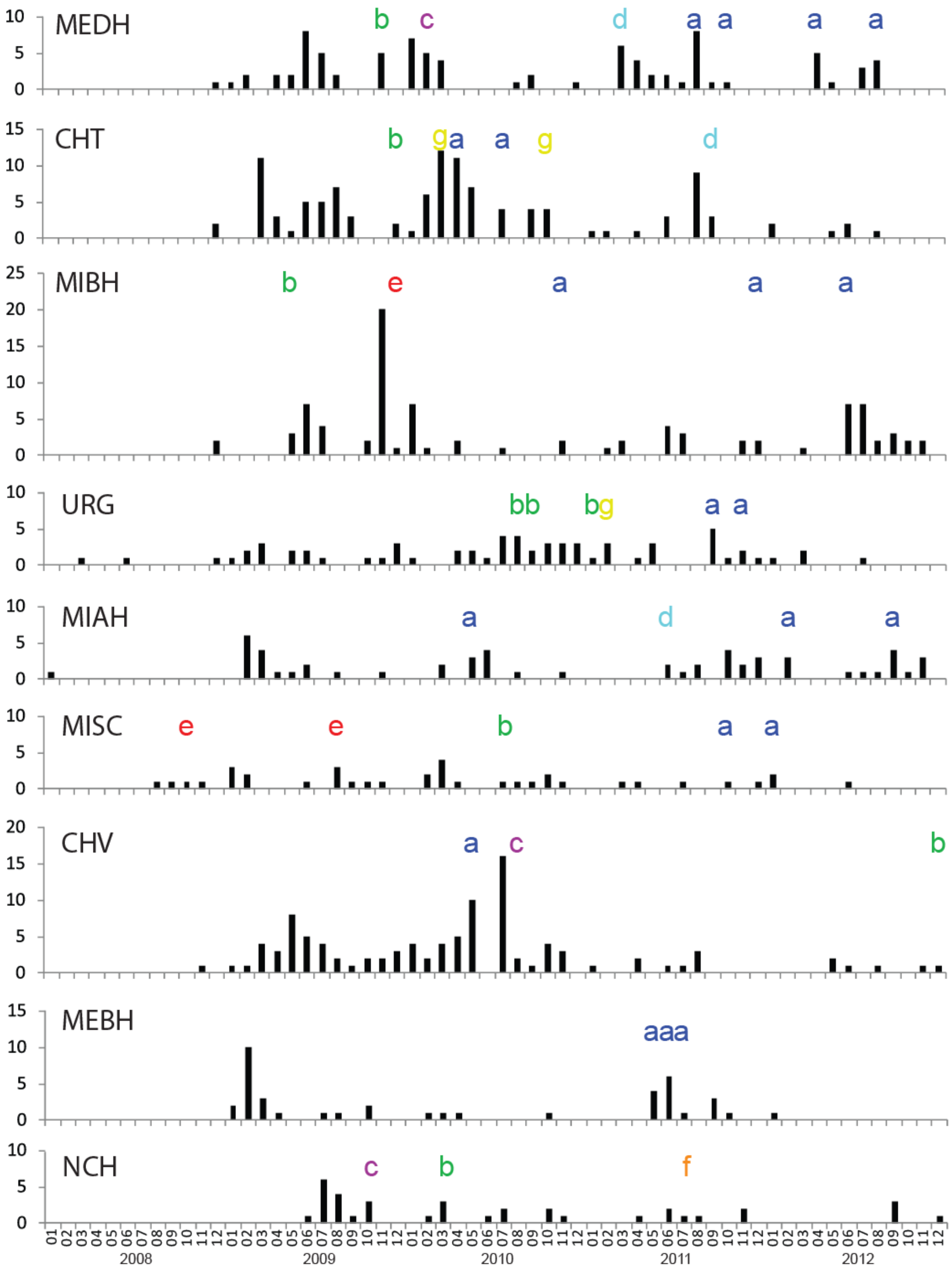

Figure S1. Continued

References

1. **Pittet D, Hugonnet S, Harbarth S, Mourouga P, Sauvan V, Touveneau S, Perneger TV.** 2000. Effectiveness of a hospital-wide programme to improve compliance with hand hygiene. Infection Control Programme. Lancet **356:**1307-1312.

2. **Blanc DS, Nahimana I, Zanetti G, Greub G.** 2013. MRSA screening by the Xpert MRSA PCR assay: pooling samples of the nose, throat, and groin increases the sensitivity of detection without increasing the laboratory costs. Eur J Clin Microbiol Infect Dis **32:**565-568.

3. **Blanc DS, Petignat C, Moreillon P, Wenger A, Bille J, Francioli P.** 1996. Quantitative antibiogram as a typing method for the prospective epidemiological surveillance and control of MRSA: comparison with molecular typing. Infect Control Hosp Epidemiol **17:**654-659.

4. **Eltringham I.** 1997. Mupirocin resistance and methicillin-resistant Staphylococcus aureus (MRSA). J Hosp Infect **35:**1-8.

5. **Basset P, Senn L, Prod'hom G, Bille J, Francioli P, Zanetti G, Blanc DS.** 2010. Usefulness of double locus sequence typing (DLST) for regional and international epidemiological surveillance of methicilin-resistant *Staphylococcus aureus*. Clin Microbiol Infect **16:**1289-1296.

6. **Basset P, Hammer NB, Kuhn G, Vogel V, Sakwinska O, Blanc DS.** 2009. *Staphylococcus aureus* clfB and spa alleles of the repeat regions are segregated into major phylogenetic lineages. Infect Genet Evol **9:**941-947.

7. Eyre DW, Golubchik T, Gordon NC, Bowden R, Piazza P, Batty EM, Ip CL, Wilson DJ, Didelot X, O'Connor L, Lay R, Buck D, Kearns AM, Shaw A, Paul J, Wilcox MH, Donnelly PJ, Peto TE, Walker AS, Crook DW. 2012. A pilot study of rapid benchtop sequencing of Staphylococcus aureus and Clostridium difficile for outbreak detection and surveillance. BMJ Open 2.

8. **Lunter G, Goodson M.** 2011. Stampy: a statistical algorithm for sensitive and fast mapping of Illumina sequence reads. Genome Res **21:**936-939.

9. Li H, Handsaker B, Wysoker A, Fennell T, Ruan J, Homer N, Marth G, Abecasis G, Durbin R. 2009. The Sequence Alignment/Map format and SAMtools. Bioinformatics 25:2078-2079.

10. **Tamura K, Stecher G, Peterson D, Filipski A, Kumar S.** 2013. MEGA6: Molecular Evolutionary Genetics Analysis version 6.0. Mol Biol Evol **30:**2725-2729.
